# Economic Evaluation Associated With Clinical-Grade Mobile App–Based Digital Therapeutic Interventions: Systematic Review

**DOI:** 10.2196/47094

**Published:** 2023-08-01

**Authors:** Yoann Sapanel, Xavier Tadeo, Connor T A Brenna, Alexandria Remus, Florian Koerber, L Martin Cloutier, Gabriel Tremblay, Agata Blasiak, Chris L Hardesty, Joanne Yoong, Dean Ho

**Affiliations:** 1 The Institute for Digital Medicine WisDM Yong Loo Lin School of Medicine National University of Singapore Singapore Singapore; 2 The N.1 Institute for Health National University of Singapore Singapore Singapore; 3 Department of Anesthesiology & Pain Medicine University of Toronto Toronto, ON Canada; 4 Department of Biomedical Engineering, College of Design and Engineering National University of Singapore Singapore Singapore; 5 Heat Resilience and Performance Centre, Yong Loo Lin School of Medicine National University of Singapore Singapore Singapore; 6 IU Internationale Hochschule GmbH Bad Honnef Germany; 7 Flying Health GmbH Berlin Germany; 8 Department of Analytics, Operations, and Information Technologies University of Quebec at Montreal Montreal, QC Canada; 9 Cytel Canada Health Inc Toronto, ON Canada; 10 Department of Pharmacology, Yong Loo Lin School of Medicine National University of Singapore Singapore Singapore; 11 Pureland Global Venture Pte Ltd Singapore Singapore; 12 Research For Impact Singapore Singapore; 13 Behavioural and Implementation Science Interventions, Yong Loo Lin School of Medicine National University of Singapore Singapore Singapore

**Keywords:** digital health, digital therapeutics, economic evaluation, cost-effectiveness, mobile phone, systematic review

## Abstract

**Background:**

Digital therapeutics (DTx), a class of software-based clinical interventions, are promising new technologies that can potentially prevent, manage, or treat a spectrum of medical disorders and diseases as well as deliver unprecedented portability for patients and scalability for health care providers. Their adoption and implementation were accelerated by the need for remote care during the COVID-19 pandemic, and awareness about their utility has rapidly grown among providers, payers, and regulators. Despite this, relatively little is known about the capacity of DTx to provide economic value in care.

**Objective:**

This study aimed to systematically review and summarize the published evidence regarding the cost-effectiveness of clinical-grade mobile app–based DTx and explore the factors affecting such evaluations.

**Methods:**

A systematic review of economic evaluations of clinical-grade mobile app–based DTx was conducted following the PRISMA (Preferred Reporting Items for Systematic Reviews and Meta-Analyses) 2020 guidelines. Major electronic databases, including PubMed, Cochrane Library, and Web of Science, were searched for eligible studies published from inception to October 28, 2022. Two independent reviewers evaluated the eligibility of all the retrieved articles for inclusion in the review. Methodological quality and risk of bias were assessed for each included study.

**Results:**

A total of 18 studies were included in this review. Of the 18 studies, 7 (39%) were nonrandomized study–based economic evaluations, 6 (33%) were model-based evaluations, and 5 (28%) were randomized clinical trial–based evaluations. The DTx intervention subject to assessment was found to be cost-effective in 12 (67%) studies, cost saving in 5 (28%) studies, and cost-effective in 1 (6%) study in only 1 of the 3 countries where it was being deployed in the final study. Qualitative deficiencies in methodology and substantial potential for bias, including risks of performance bias and selection bias in participant recruitment, were identified in several included studies.

**Conclusions:**

This systematic review supports the thesis that DTx interventions offer potential economic benefits. However, DTx economic analyses conducted to date exhibit important methodological shortcomings that must be addressed in future evaluations to reduce the uncertainty surrounding the widespread adoption of DTx interventions.

**Trial Registration:**

PROSPERO International Prospective Register of Systematic Reviews CRD42022358616; https://www.crd.york.ac.uk/prospero/display_record.php?ID=CRD42022358616

## Introduction

### Background

The continued rise in chronic and mental health conditions, and commensurately in their associated health care costs, is not a new phenomenon. What is new—and reinforced by the COVID-19 pandemic—is the realization of a need for novel approaches to deliver care for these conditions closer to where individuals live and work, such as in their own homes and communities. As health care organizations and providers rush to adapt to this new reality, the adoption of digital technologies has accelerated rapidly [1].

Under the umbrella term digital technologies, it is crucial to distinguish between 3 separate categories, which are sometimes conflated or used interchangeably: *wellness and support* solutions, referring to products designed to capture, store, and transmit health data (eg, telehealth platforms); *diagnostic and monitoring* solutions, involving products that measure or track individuals’ health status or both (eg, connected drug delivery devices); and *digital therapeutics (DTx)*, a new class of medicine that delivers therapeutic interventions directly to patients (eg, digital behavioral therapy) [2].

Powered by computer software, DTx can deliver evidence-based therapeutic interventions that prevent, manage, or treat a spectrum of medical disorders and diseases directly to patients [2]. Evidence supporting the potential of DTx in optimizing patient care and health outcomes [3] through a more personalized approach to health care, with greater patient education and empowerment, is mounting [4]. As such, DTx have recently been described as the “next paradigm” of modern health care [5].

Interest in DTx began to surge in 2017 when the US Food and Drug Administration approved the first DTx for the treatment of opioid use disorders [6]. Subsequently, in 2019, Germany became the first country to establish a Fast-Track Process for integrating DTx into the German reimbursement market [7]. Shortly thereafter, Belgium [8], France [9], Japan [10], South Korea [11], and the United Kingdom [12] began to implement DTx-specific approval and reimbursement processes. Globally, there are currently approximately 400 DTx available or under development [13].

Although considered a rapidly emerging class of medicine, the economic value of DTx is yet to be understood, resulting in an important knowledge gap that limits its widespread uptake [14,15]. There is currently limited consensus on whether these technologies are cost-effective compared with traditional treatments. Because cost-effectiveness is an important consideration in payers’ reimbursement and pricing decisions [15], questions regarding the potential economic impact of DTx merit exploration [16].

In the context of budgetary constraints and the enduring need for optimal resource allocation in health care, determining the best mix of health services and treatments to maximize clinical outcomes while minimizing costs is critical [17,18]. If DTx can demonstrate its economic value to decision makers (eg, public and private payers, regulators, and care providers), such evidence is important to facilitate decisions around market access, pricing, and reimbursement (and, therefore, adoption) for these technologies [19]. Therefore, we sought to systematically answer the question of whether this recently emerged class of medical intervention, DTx, has yet been translated to economic value.

### Objective

Given the growing body of evidence supporting the potential clinical benefits of DTx, the aim of this systematic review was to evaluate the published evidence regarding the cost-effectiveness of clinical-grade, mobile app–based DTx interventions and explore the costs and factors that drive such economic evaluation (EE).

## Methods

### Search Strategy

The protocol for this review was registered with PROSPERO a priori (CRD42022358616). A search of the relevant literature was performed in accordance with the PRISMA (Preferred Reporting Items for Systematic Reviews and Meta-Analyses) 2020 guidelines [20]. Databases searched for eligible studies included PubMed, Cochrane Library, Web of Science, Embase, Business Source Ultimate (EBSCO), CINAHL (EBSCO), Scopus, ProQuest Business Premium Collection, and the Wiley Online Library. The search was conducted between September 5, 2022, and October 28, 2022, and was not constrained by the year of publication. In addition, secondary searches were executed in the International Network of Agencies for Health Technology Assessment International Health Technology Assessment database and the *International Journal of Technology Assessment in Health Care*. The search strings were tailored according to each database requirement. The following keywords were searched in publication titles and abstracts, as identified by the setting, perspective, intervention, comparison, and evaluation framework [21] and in consultation with a research librarian from the National University of Singapore:

(A): “digital therapeutic*” OR “digital health*” OR “digital tech*” OR “mobile health” OR “mhealth” OR “mobile tech*” OR “mobile medical app*” OR “mobile app*” OR “wearable tech*” OR “connected medical devices”; AND

(B): “economic evaluation” OR “economic value” OR “cost-benefit” OR “cost-utility” OR “cost-effectiveness” OR “cost-effective” OR “Quality-Adjusted Life-Years” OR “Markov Chains” OR “Models, Economic.”

### Eligibility Criteria

DTx delivery mechanism (eg, mobile apps, web-based systems, or virtual reality) can significantly impact its economic proposition. Therefore, because DTx primarily leverage mobile apps as a delivery mechanism [22], and “smartphone apps” are regarded as the top 2 (after telemedicine) technology developments anticipated to create the most disruption for established health care practices [23]; hence, this review focuses on clinical-grade mobile app–based DTx. Thus, studies were included based on the following inclusion criteria: (1) published in a peer-reviewed journal within any time frame, (2) the study analyzed a mobile app–based intervention, (3) the therapeutic intervention was delivered directly to patients, (4) the intervention demonstrated its clinical benefits through at least 1 case-control study, (5) the study included a partial or full EE, and (6) the publication was available in English. Internet-based and virtual reality–based interventions, solutions for screening, diagnostic and monitoring purposes, telemedicine and remote patient monitoring solutions, and clinical decision support solutions were excluded. Furthermore, non–peer-reviewed publications (eg, white papers and editorials), abstract-only papers, and those with unavailable full text were also excluded.

The reference lists of studies that met the inclusion criteria were subjected to an additional “backward reference search” to identify additional relevant studies.

### Study Selection, Data Extraction, and Data Synthesis

After duplicate records were removed, 2 reviewers (YS and XT) independently screened the titles and abstracts of all remaining identified studies for inclusion using the systematic review software Covidence (Veritas Health Innovation). Eligible studies that met the inclusion criteria, according to both reviewers, then underwent a full-text review. Conflicting outcomes were discussed between reviewers, and a third researcher (AR) was involved to help reach a consensus when necessary.

Data were extracted using a bespoke web-based Microsoft Excel 365 spreadsheet. Full data extraction was completed by 1 reviewer (YS) and verified by a second reviewer (XT). The extracted information from each study included country, targeted disease, product’s primary purpose, study design, perspective, costs considered, time horizon, intervention group sample size, type of control group, clinical outcomes, cost savings, scholars’ conclusion on the intervention’s cost-effectiveness, uncertainty consideration (discounting and sensitivity analysis), and sources of funding or conflicts of interest. Additional factors directly considered in the EE and factors reported by scholars as impacting the DTx’s economic impact, through sensitivity analysis or explicitly in the studies’ discussion sections, were also extracted. After extraction, the data were narratively synthesized to evaluate their meaning [24]. The additional extracted factors impacting the DTx were clustered into main categories and organized into a concept matrix [25].

### Quality Assessment

Quality assessment of the included studies was conducted using the Consensus Health Economic Criteria (CHEC) list [26]. Each study received a score of 1, 0.5, or 0 for satisfying, partially satisfying, or not satisfying, respectively, the 19 independent evaluation criteria. The cumulative percentage of criteria satisfied was calculated as an overall “score” for each article (maximum possible score: 19/19 criteria or 100%).

The risk of bias (RoB) was calculated for each article according to its methodology. For randomized controlled trials (RCTs), the Cochrane Collaboration RoB tool [27] was used, rating each study as unclear, low, or high risk for selection bias. For nonrandomized studies, the Risk of Bias in Non-Randomized Studies of Interventions tool was used to rate the RoB owing to confounding, bias in selection of participants into the study, bias in the classification of interventions, bias owing to deviation from intended interventions, bias owing to missing data, bias in measurement outcomes, and bias in selection of the reported result [28]. Each of these features was rated as low, moderate, or serious RoB, and each study’s overall bias was conservatively calculated as the highest-risk measure in any category. Finally, bias in modeling studies was calculated using the Bias in Economic Evaluation checklist, and rated as “Yes,” “No,” “Partially,” “Unclear,” or “Not applicable” referring to a study’s ability to address each of 22 independent criteria [29]. We elected to consolidate the Bias in Economic Evaluation ratings into the scale’s 4 overarching categories: overall checklist for bias in EE, bias related to structure, bias related to data, and bias related to consistency. For uniformity with the other RoB assessment tools, we rated the bias in each category as low, moderate, or high risk, equivalent to the highest-risk single evaluation for any component criterion, considering “Yes” and “Not applicable” to be equal to low risk, “Partially” and “Unclear” to be equal to moderate risk, and “No” to be equal to high risk.

## Results

### Study Selection

**Figure 1:**
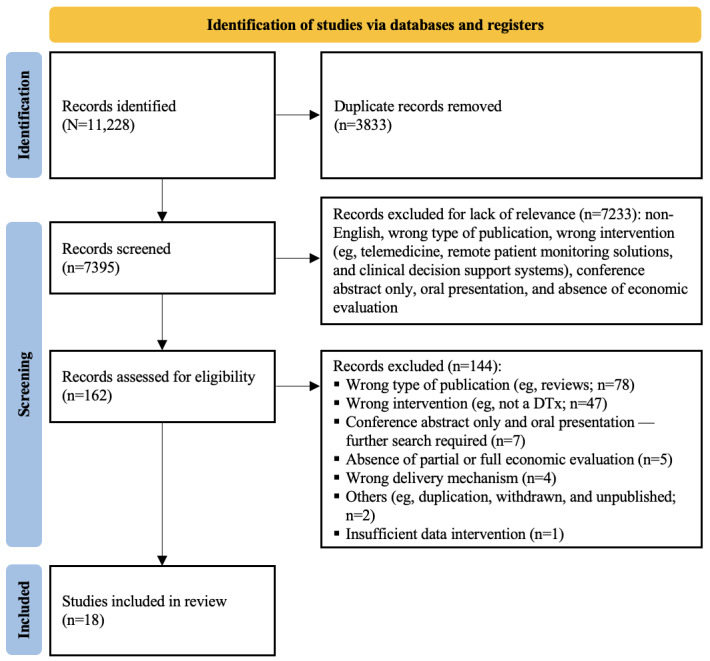
PRISMA (Preferred Reporting Items for Systematic Reviews and Meta-Analyses) flow diagram. DTx: digital therapeutics.

After duplicate removal and eligibility screening, 18 studies were included in this review (Figure 1).

### Study Characteristics

Table 1 summarizes the characteristics and main health economic outcomes associated with the included studies. Overall, the 18 studies in this review were conducted between 2016 and 2022. Of the 18 studies, 10 (56%) [30-39] were conducted in the United States; 2 (11%) [40,41] in the Netherlands; 2 (11%) [42,43] in Sweden; 1 (6%) [44] in Germany; 1 (6%) [45] in Japan; 1 (6%) [46] in the United Kingdom; and 1 (6%) [47] jointly in the Netherlands, Spain, and Taiwan. Furthermore, of the 18 studies, 10 (56%) [30-32,34-39,45] were industry funded, 6 (33%) [40,41,43,44,46,47] were publicly funded, and the remaining 2 (11%) [33,42] received mixed funding.

The targeted diseases for DTx in the included studies, all among adult patient populations, were urinary incontinence (3/18, 17%) [40,42,43], diabetes (2/18, 11%) [32,36], opioid use disorder (2/18, 11%) [34,35], hypertension (2/18, 11%) [45,46], generalized anxiety disorder (1/18, 6%) [37], chronic insomnia (1/18, 6%) [31], osteoarthritis (1/18, 6%) [41], lower back pain (1/18, 6%) [44], obesity (1/18, 6%) [30], behavioral health conditions (1/18, 6%) [33], cardiovascular disease (1/18, 6%) [47], both diabetes and cardiovascular disease (1/18, 6%) [39], and both type 2 diabetes and hypertension (1/18, 6%) [38].

Regarding the type of EE performed, of the 18 studies, 7 (39%) [30-36] involved nonrandomized study–based EE, 6 (33%) [37-39,44,45,47] involved model-based EE, and 5 (28%) [40-43,46] involved RCT-based EE. Of the 18 studies, 12 (67%) used a payer perspective [30-36,38,39,41,45,46], whereas 6 (33%) used a societal perspective [37,40,42-44,47], with 2 (11%) of the latter group also taking a payer perspective [37,47]. The time horizon used for the EE was between 6 and 12 months for 56% (10/18) of the studies [32-36,40-43,46], 24 months for 6% (1/18) of the studies [31], 36 months for 17% (3/18) of the studies [30,38,44], 60 months for 6% (1/18) of the studies [47], 120 months for 6% (1/18) of the studies [39], and lifetime for 11% (2/11) of the studies [37,45]. The intervention group sample sizes ranged between 60 and 305 participants for RCT-based EE and between 248 and 4790 participants for nonrandomized study–based EE. The interventions were compared with usual care (13/18, 72%) [30,32-34,36,38,40,41,43-47], preintervention (2/18, 11%) [31,39], an informative but noninterventional “control” app (1/18, 6%) [42], patients who filled their prescription but did not engage beyond week 1 and patients who did not fill the prescription (1/18, 6%) [35], and traditional cognitive behavioral therapy or no therapy (1/18, 6%) [37].

Of the 18 studies, 14 (78%) [30-32,37-47] assessed the impact of the DTx intervention on clinical outcomes. Of the 14 studies, 11 (79%) [30-32,37-39,42-46] found superior clinical outcomes, 2 (14%) [40,41] found no improvement compared with usual care, and 1 (7%) [47] found superior clinical outcomes in only 1 of the 3 countries in which the intervention under study was delivered.

Half (9/18, 50%) of the studies included in this review [37,38,40-42,44-47] conducted a cost-effectiveness analysis (CEA), with 4 (22%) also including a cost-utility analysis (CUA) [40-42,44]. Of the 18 studies, 8 (44%) conducted a cost analysis [30-36,39], with a strong emphasis on cost differences using, for example, pre-post intervention claims data, and 1 (6%) study focused solely on CUA [43]. Of the 10 studies using CEA and CUA methods, 7 (70%) presented incremental cost-effectiveness ratio (ICER) values based on the cost per quality-adjusted life year (QALY) gained to assess the cost-effectiveness of the DTx intervention [37,38,42-45,47]. Meanwhile, 20% (2/10) of the CEA and CUA studies showed ICER values based on cost per incontinence impact–adjusted life years gained and cost per mm Hg reduction in blood pressure [40,46]. In total, 10% (1/10) of the studies did not report an ICER but an incremental net monetary benefit [41].

**Figure 2:**
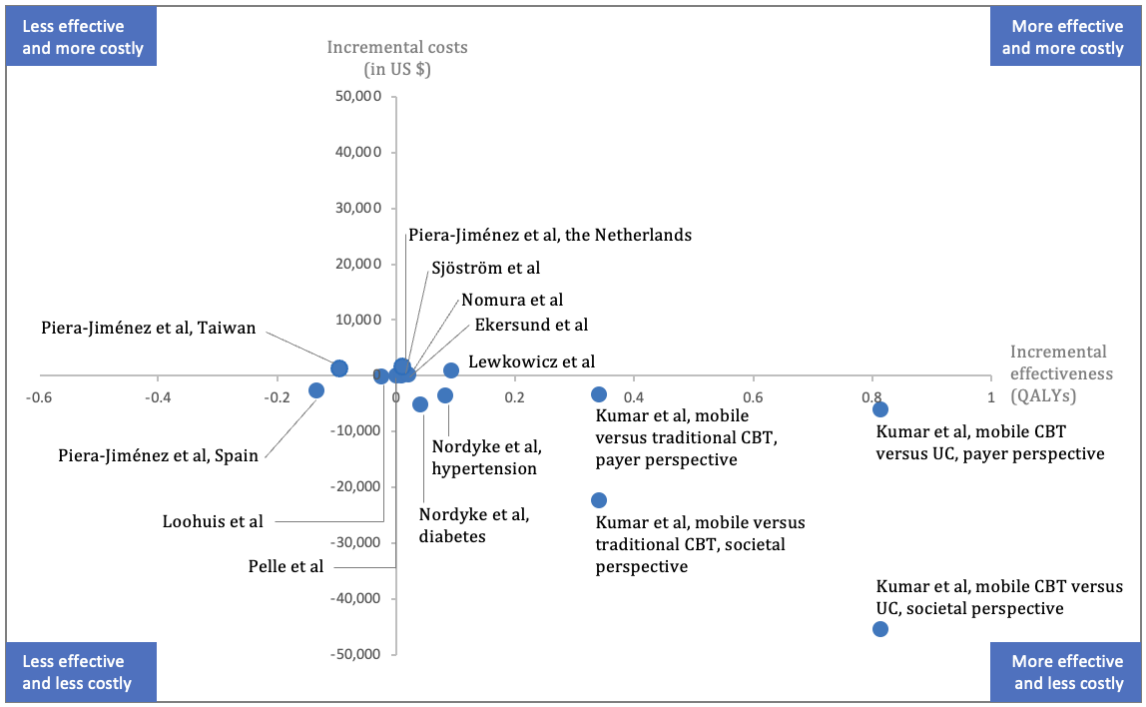
Cost-effectiveness plane of studies with cost and quality-adjusted life years as the outcome measures [37,38,40-45,47]. CBT: cognitive behavioral therapy; QALY: quality-adjusted life year; UC: usual care.

Of the 10 studies that conducted a full EE, 9 (90%) [37,38,40-46] found the DTx intervention to be cost-effective in the context of the study, whereas 1 (10%) study found the intervention to be cost-effective in only 1 of the 3 countries in which it was studied [47]. Specifically, DTx accounted for QALY gains along with cost savings in 20% (2/10) of the studies [37,38], QALY gains along with higher costs at an acceptable ICER in 50% (5/10) of the studies [34,35,42,43,46], QALY losses with cost savings in 20% (2/10) of the studies [40,47], and no demonstrable effectiveness difference with cost savings in 20% (1/10) of the studies [41]. Figure 2 represents the 15 different DTx interventions under assessment in the 50% (9/18) of the studies [37,38,40-45,47] that reported cost and QALYs as the outcome measures across the quadrants of the cost-effectiveness plane. The horizontal axis of the plane indicates differences in effects (ie, health outcomes), whereas the vertical axis represents the differences in costs between the DTx interventions and their respective comparators.

Of the 8 studies based on partial EE, all 8 (100%) found the DTx intervention under evaluation to be cost saving [30-36,39].

**Table 1:** Study characteristics.

Study; country			Targeted disease (categories of DTx^a,b^)		Type of evaluation		Perspective		Time horizon (months)		Intervention group sample size		Intervention vs comparator		Did the intervention lead to superior clinical outcomes?		Did the intervention lead to cost savings (in US $)?		Is the intervention cost-effective (incremental cost-effectiveness ratio, in US $)?		Consensus Health Economic Criteria (%)
**Randomized clinical trial–based economic evaluations**																					
	Ekersund et al [42], 2022; Sweden	Urgency and mixed urinary incontinence (manage)		CEA^c^ and CUA^d^		Societal		12		60		Tät II vs information app		Yes (0.0115 QALY^e^ gained)		No (+144)		Yes (12477/QALY)		92	
	Pelle et al [41], 2022; NL^f^	Osteoarthritis (manage)		CEA and CUA		Health care payer		6		214		Dr Bart vs UC^g^		No difference		Yes (−23)		Yes (56 iNMB^h^)		76	
	McManus et al [46], 2021; United Kingdom	HTN^i^ (manage)		CEA		NHS^j^ payer		12		305		Home and Online Management and Evaluation of Blood Pressure vs UC		Yes (a mean difference in SBP^k^ of −3.4mm Hg, and −0.5 mm Hg in DBP^l^)		No (+46)		Yes (13/mm Hg reduction)		100	
	Sjöström et al [43], 2017; Sweden	Stress urinary incontinence (manage)		CUA		Societal		12		62		Tät vs UC		Yes (0.00849 QALY gained)		No (+69)		Yes (8071/QALY)		95	
	Loohuis et al [40], 2022; NL	Stress, urgency, or mixed urinary incontinence (manage)		CEA and CUA		Societal		12		131		URinControl vs UC		No (0.025 QALY loss and 0.043 IIALY^m^ gained)		Yes (−170)		Yes (−3918/IIALYs)		87	
**Nonrandomized study–based economic evaluations**																					
	Horstman et al [30], 2021; United States	Overweight and obesity (manage)		CA^n^		Payer		36		4790		Real Appeal vs UC		Yes (3% greater weight loss on average per participant)		Yes (−771/participant)		—^o^		55	
	Forma et al [31], 2022; United States	Chronic insomnia (treat)		CA		Payer		24		248		Pre-post Somryst treatment intervention		Yes (37.2% insomnia severity index score declined/participant)		Yes (−2059/participant)		—		39	
	Sweet et al [32], 2020; United States	Diabetes (prevent)		CA		Employer and payer		12		2027		Omada vs UC		Yes (4.3% average weight loss)		Yes (−1169/participant)		—		55	
	Abhulimen et al [33], 2018; United States	Behavioral health condition^p^ (manage)		CA		Public and payer		11		799		myStrengh vs UC		—		Yes (−382/participant)		—		53	
	Velez et al [34], 2022; United States	OUD^q^ (treat)		CA		Payer		12		901		reSET-O vs UC		—		Yes (−2791/participant)		—		63	
	Velez et al [35], 2021; United States	OUD (treat)		CA		Payer		9		444		reSET-O vs nonengagers		—		Yes (−2708/participant)		—		50	
	Whaley et al [36], 2019; United States	Diabetes (manage)		CA		Employer and payer		12		2261		Livongo program vs UC		—		Yes (−1056/participant)		—		55	
**Model-based economic evaluations**																					
	Piera-Jiménez et al [47], 2020; NL, Spain, and TW^r^	CVD^s^ (prevent)		CEA (RCT^t^ informed and Markov model)		Societal and health care payer		60		120		Do change 2 vs UC		NL: yes (0.011 QALY gained); Spain: no (0.134 QALY loss); TW: no (0.094 QALY loss)		NL: no (+1456); Spain: yes (−2666); TW: no (+1127)		NL: no (131959/QALY); Spain: yes (19895/QALY); TW: no		76	
	Lewkowicz et al [44], 2022; Germany	Low back pain (manage)		CEA and CUA (RCT informed and Markov model)		Societal		36		RCT: 53 model: 10,000		Kaia vs UC		Yes (0.0221 QALY gained)		No (+129)		Yes (5815/QALY)		87	
	Nomura et al [45], Japan, 2022	Hypertension (treat)		CEA (RCT informed and Markov model)		Public health care payer		Lifetime		199		CureApp and UC vs UC		Yes (0.092 QALY gained)		No (+962)		Yes (10434/QALY)		92	
	Kumar et al [37], 2018; United States	Generalized anxiety disorder (prevent/treat)		CEA (pilot study informed and Markov model)		Societal and payer		Lifetime		Pilot: 89 model: 100,000		Mobile CBT^u^ vs traditional CBT (model A) and mobile CBT vs UC (model B)		Model A: yes (34,108 QALYs gained); model B: yes (81,492 QALYs gained)		Societal: model A: yes (−2.23 billion); model B: yes (−4.54 billion); payer: model A: yes (−339 million); model B: yes (−605 million)		Societal: model A: yes (−65380/QALY); model B: yes (−55710/QALY); payer: model A: yes (−9939/QALY); model B: yes (−7424/QALY)		74	
	Nordyke et al [38], 2019; United States	Diabetes and HTN (manage)		CEA (decision tree model)		US commercial payer		36		—		DTx+UC vs UC		T2DM^v^: yes (0.0427 QALY gained); HTN: yes (0.0827 QALY gained)		T2DM: yes (−5220); HTN: yes (−3480)		T2DM: yes (−122,248/QALY); HTN: yes (−42,080/QALY)		55	
	Chen et al [39], 2016; United States	Diabetes and CVD (prevent)		CA (best available evidence and Markov model)		Public and payer		120		1121		Pre-post Omada program intervention		Yes (6.8% reduction in body weight per participant)		Yes (from 11,550 to 14,200 per participant)		—		76	

^a^DTx: digital therapeutics.

^b^Classified as “manage” medical disorders and conditions (eg, manage chronic conditions that can be controlled but not cured, including symptoms management), “treat” (eg, toward permanent recovery, such as for addictions and chronic insomnia), or “prevent” (eg, secondary prevention of cardiovascular diseases).

^c^CEA: cost-effectiveness analysis.

^d^CUA: cost-utility analysis.

^e^QALY: quality-adjusted life year.

^f^NL: the Netherlands.

^g^UC: usual care.

^h^iNMB: incremental net monetary benefit, which is easier to interpret than the incremental cost-effectiveness ratio when differences are small [41].

^i^HTN: hypertension.

^j^NHS: National Health Service.

^k^SBP: systolic blood pressure.

^l^DBP: diastolic blood pressure.

^m^IIALY: incontinence impact–adjusted life years.

^n^CA: cost analysis.

^o^Not available.

^p^Including depression, anxiety, insomnia, and substance use disorders.

^q^OUD: opioid use disorder.

^r^TW: Taiwan.

^s^CVD: cardiovascular disease.

^t^RCT: randomized controlled trial.

^u^CBT: cognitive behavioral therapy.

^v^T2DM: type 2 diabetes mellitus.

### Quality Assessment

The level of methodological detail presented in the included studies varied but was overall high. The mean study quality score, determined using the CHEC list, was 71% (SD 0.18%; Multimedia Appendix 1 [30-47]). Quality was the highest among EE based on RCTs and the lowest among those based on nonrandomized studies (Multimedia Appendix 1). Common areas for point deduction included no mention of ethical and distributional considerations, the single most common quality issue, limited descriptions of interventions’ alternatives (ie, of any interventions received by control groups), unjustified decisions to use narrow methodological perspectives (eg, health care resource use rather than societal perspectives), a lack of incremental analysis of costs and outcomes (ie, an ICER), and no discussion of generalizability to other settings and patient populations. In contrast, most of the included studies measured and valued outcomes appropriately, clearly described study populations, and explicitly acknowledged the potential conflicts of interest. Study quality was not significantly associated with the year of publication.

Although all RCT-based EE (5/5, 100%) performed sensitivity analyses (ie, univariate and multivariate scenarios as well as one-way and multiway deterministic sensitivity analyses), only 29% (2/7) of the nonrandomized study–based EE [33,34] did so (ie, multiway deterministic sensitivity analyses). In total, 83% (5/6) of model-based EE [37-39,44,45] performed sensitivity analyses (ie, one-way and multiway deterministic sensitivity analyses as well as probabilistic sensitivity analyses).

RoB was similarly heterogeneous for RCTs, nonrandomized studies, and modeling studies, with important overall risks of bias across studies. None of the RCTs were classified as having a low RoB in any of the 6 categories. Owing to the digital nature of the DTx, participants (or personnel) were not blind to assignment and could therefore expect to receive either an active treatment or a placebo. Consequently, most RCT-based studies (4/5, 80%) [40,41,43,46] were classified as having a high risk of performance bias. One study used an “information app” for the comparator group, and without clear consequence on the potential performance bias, it was classified as unclear [42]. In total, 40% (2/5) of the studies [40,41] were classified as high risk for attrition bias related to incomplete outcome data resulting from a high degree of participant attrition that was not fully accounted for in the analyses (Multimedia Appendix 2 [40-43,46]). Common sources of “other bias” were potential selection biases in participant recruitment, leading to potential imbalance between groups in baseline variables (such as age, educational level, or disease severity).

Among the nonrandomized studies, none received an overall low-risk classification (Multimedia Appendix 3 [30-36]): 57% (4/7) [30,32,33,36] were graded as moderate risk and 42% (3/7) [31,34,35] as high risk, in all cases owing to moderate- or high-risk classifications in 1 to 3 (out of 7) scoring categories. All nonrandomized studies demonstrated a low RoB in the classification of interventions, potential deviation from intended interventions, measurement of outcomes, and selection of reported results. The greatest source of potential bias among these nonrandomized studies was the selection of participants in the study, as patients often self-selected or were recruited into intervention groups based on potentially confounding factors. For example, 2 studies of the opioid abstinence tool reSET-O were graded as serious risk in this category because the intervention group comprised patients who sought a reSET-O prescription or filled one provided by a prescriber, whereas the control group comprised patients who did not actively seek treatment in the same way [34,35].

Finally, among the modeling studies, RoB was classified as unclear in the category of bias related to internal consistency in all 6 studies, none of which explicitly reported exploring this (Multimedia Appendix 4 [37-39,44,45,47]). The highest RoB among these studies tended to arise from part A of the checklist: the “overall checklist for bias in economic evaluation.” Within this category, 66% (4/6) [37-39,45] of the studies were graded as high RoB and 16% (1/6) [44] as moderate RoB. Common sources of potential bias among modeling studies were narrow perspectives without justification, a lack of ordinal ICER, and a short time horizon relative to the outcome of interest.

### Costs and Factors Impacting the Economic Value of DTx

#### Overview

The costs and factors associated with the economic impact of the DTx interventions, which were obtained through sensitivity analysis or outlined in the discussion sections of the individual studies as having an impact on DTx economic value, were extracted from the selected studies (Table 2). These costs and factors reflect, above all, the medical conditions and disorders under consideration as well as the study design and methods for measuring economic outcomes. Nevertheless, there are some common key findings that are worth noting.

**Table 2:** Costs and factors impacting the economic value of digital therapeutics (DTx).

		Societal-perspective studies							Payer-perspective studies											
		[40]	[43]	[42]	[37]	[47]	[44]	[32]		[36]	[39]	[30]	[46]	[45]	[38]	[34]	[35]	[41]	[31]	[33]
**Direct medical and nonmedical costs**																				
	Pharmaceutical treatment^a^	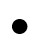 ^b^	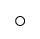 ^c^	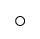	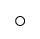	—^d^	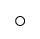	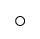		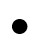	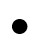	—	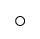	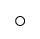	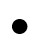	—	—	—	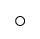	—
	Cost of the DTx	—	—	—	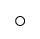	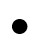	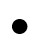	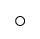		—	—	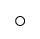	—	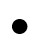	—	—	—	—	—	—
	HRU^e^: primary care^f^	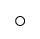	—	—	—	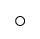	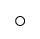	—		—	—	—	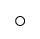	—	—	—	—	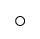	—	—
	HRU: outpatient care^g^	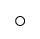	—	—	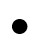	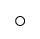	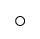	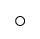		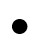	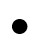	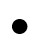	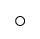	—	—	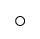	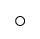	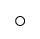	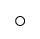	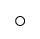
	HRU: inpatient care^h^	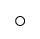	—	—	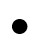	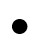	—	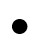		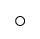	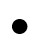	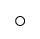	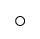	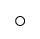	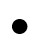	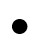	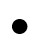	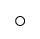	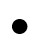	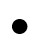
	HRU: ED^i^ visits	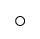	—	—	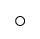	—	—	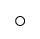		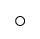	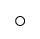	—	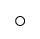	—	—	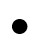	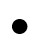	—	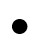	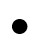
	HRU: health support intervention^j^	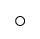	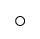	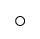	—	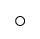	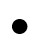	—		—	—	—	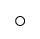	—	—	—	—	—	—	—
	Intervention-specific training^k^	—	—	—	—	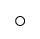	—	—		—	—	—	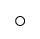	—	—	—	—	—	—	—
	Participants’ time spent on the DTx^l^	—	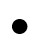	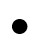	—	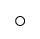	—	—		—	—	—	—	—	—	—	—	—	—	—
**Indirect medical and nonmedical costs**																				
	Productivity impact^m^	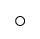	—	—	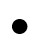	—	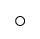	—		—	—	—	—	—	—	—	—	—	—	—
	DTx maintenance	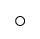	—	—	—	—	—	—		—	—	—	—	—	—	—	—	—	—	—
**Influencing factors**																				
	Participants’ baseline characteristics^n^	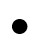	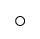	—	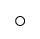	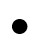	—	—		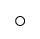	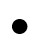	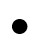	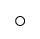	—	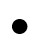	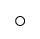	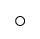	—	—	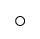
	Reimbursement rate	—	—	—	—	—	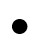	—		—	—	—	—	—	—	—	—	—	—	—
	Treatment adherence	—	—	—	—	—	—	—		—	—	—	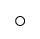	—	—	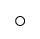	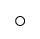	—	—	—
	Attrition rate^o^	—	—	—	—	—	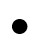	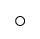		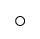	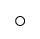	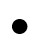	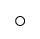	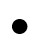	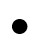	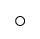	—	—	—	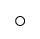
	Degree of clinical inertia	—	—	—	—	—	—	—		—	—	—	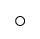	—	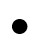	—	—	—	—	—
	Sustained DTx clinical effectiveness^p^	—	—	—	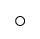	—	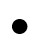	—		—	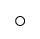	—	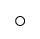	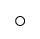	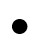	—	—	—	—	—

^a^Including core costs originating from spending related to treatment of medical disorders and diseases, such as diabetes, or materials and aids, as in the case of incontinence.

^b^Factors considered and directly cited by researchers as impacting the cost-effectiveness of DTx.

^c^Factors reported by researchers as having an “important” or “significant” impact on, or which were deemed as “decisive” to, the cost-effectiveness of DTx.

^d^Not applicable.

^e^HRU: health care resource use.

^f^General practitioners, physical therapists, occupational therapists, exercise therapists, dieticians, or other primary care practitioners.

^g^Outpatient or ambulatory care visits, medical specialist consultations, physician services, and pathology and laboratory services.

^h^Including partial hospitalizations.

^i^ED: emergency department.

^j^Health assistance interventions and support provided by health care workers.

^k^Training and educational sessions related to the optimal implementation of the intervention, including in-person or web-based sessions, for either patients or clinicians.

^l^Costs associated with the time spent by study participants using the DTx.

^m^Productivity losses such as absenteeism and disability days.

^n^Demographic and risk factor profiles of the study participants, such as race, age, gender, ethnicity, disease evolution, and severity or presence of comorbidities.

^o^Including study participants’ engagement level with the DTx.

^p^Medium- to long-term relative effectiveness of the DTx intervention, including its effect on preventing or delaying disease onset.

#### Health Care Resource Use

Health care resource use, which includes primary care, outpatient care, inpatient care, emergency department visits, and health support intervention, was the most frequently examined and reported cost across studies (18/18, 100%) [30-47]. Of the 18 studies, inpatient care–related costs were shown to have a potential impact on the economic impact of DTx in 15 (78%) studies [30-41,45-47]. Inpatient care was further categorized as a “decisive factor” (ie, having a significant impact on the economic impact of DTx [37]) in half (9/18, 50%) of the studies [30-35,37-39].

#### Pharmaceutical Treatment

The expenditures originating directly from treating medical conditions or disorders, such as those related to the consumption of drugs (eg, frequency and dose) or materials and aids, were considered in 67% (12/18) of the studies [31,32,36-40,42-46].

In some cases, DTx interventions have been shown to improve treatment adherence [34,35] and, as a result, might increase some expenses such as overall drug therapy costs or costs associated with higher rates of use of certain clinician services (eg, psychiatry services, outpatient visits, and pathology or drug testing). However, in many cases, these expenses were largely compensated by the cost savings in the included studies, especially in health care resource use [34,35]. For example, in a trial by McManus et al [46], participants who used DTx were more likely to have their antihypertensive drugs adjusted during the study (ie, dosage or change in drugs).

In other cases, the clinical benefits of DTx treatments may be able to reduce or eliminate the need for pharmacotherapies, thereby lowering total medical expenditures. However, Nordyke et al [38] and McManus et al [46] noted that despite evidence of DTx clinical efficacy, there may be a delay in deprescribing drugs from health care professionals—a phenomenon known as clinical inertia, which may reduce the potential economic benefits of DTx.

#### Participants’ Baseline Characteristics

More than half (12/18, 67%) of the studies [30,33-40,43,46] pointed out that participants’ baseline characteristics, such as age, gender, ethnicity, education level, baseline disease severity, risk factors, and costs, had an impact on the economic value of the intervention. Loohuis et al [40] conducted a subgroup analysis that revealed differences in DTx effects and costs not only by disease severity but also by recruitment type: participants recruited via social media incurred lower associated costs and experienced a lesser treatment effect than those recruited by a general practitioner.

Whaley et al [36] hypothesized that individuals with higher health care needs who accepted the program invitation generally had higher baseline levels of comorbidity and health care spending than those who did not enroll and therefore were more motivated to try a new intervention and more likely to voluntarily enroll in a digital intervention. Piera-Jiménez et al [47] also noted that the willingness of individuals to adopt an intervention strongly impacts the success of a DTx intervention.

#### Attrition Rate

More than half (10/18, 56%) of the studies [30,32-34,36,38,39,44-46] considered the potential causal effect of attrition rate, which can be a critical factor [44], on the DTx economic impact. Discrepancies in the manner in which such factors were evaluated should be noted. First, as highlighted by Lewkowicz et al [44], a DTx intervention’s attrition rates in RCT-based EE might simply not be reported or may not “represent real-world engagement and program dropout rates.” Second, some studies defined a minimum level of engagement for participants’ data to be included for extraction and analysis; for example, in the study by Pelle et al [41], an RCT-based EE, 63 participants were excluded for suboptimal level of engagement. Finally, in claims-based EE, the impact of a DTx intervention was evaluated based on a patient population that, by definition, filled their prescription and engaged with the therapeutic, which might have led to bias in the selection of participants in the study (Multimedia Appendix 4). As Nomura et al [45] highlighted, “achieving good cost-effectiveness for DTx might require sensitive handling to balance the appropriate DTx app usage duration with DTx costs and expected attrition rate.” The attrition rate may have also resulted in incomplete outcome data, a potential RoB in half of the RCT and nonrandomized study–based EE (Multimedia Appendices 2 and 3) [31-34,40,41].

## Discussion

### Main Findings

The EE of new therapies and clinical interventions is critical for market access and adoption because they provide decision makers with important information regarding their “value for money.” This systematic review included 18 studies that evaluated the EE of clinical-grade, mobile app–based DTx. The relatively small number of included studies (which is consistent with other recent systematic reviews of digital health solutions [48]) attests to the paucity of published literature on DTx, which also explains the scarcity of evidence pertaining to the economic value of these intervention modalities [49].

All 18 included studies were conducted in high-income countries, with 12 supported by industry funding [30-39,45,46] and 6 by public organizations [40-44,47]. Although the prevalence of industry-funded research may potentially introduce commercial bias, which is acknowledged in this review, it also underscores the contributions of both public and private organizations in generating evidence for informed treatment decision-making when DTx options are available.

### Heterogeneity Among Included Studies

The included studies exhibited significant heterogeneity with respect to DTx intervention, type of EE, and methodology (Table 1). This review combines EEs based on both clinical trial results and decision modeling to examine DTx applications for a spectrum of diseases across various settings and for different payers. Specifically, 4 studies [33-36] did not report the clinical outcomes of the intervention, only 10 reported an ICER [37,38,40-47], and only 7 reported cost and QALYs as the outcome measures [37,38,42-45,47]. As a result of this heterogeneity, a robust meta-analysis of these data was not feasible, making it impossible to provide numerical answers regarding the cost-effectiveness of DTx interventions. The study heterogeneity also hinders the comparability and generalizability of the findings and makes the EE results difficult to interpret.

In addition to this challenge, the context-specific nature of DTx interventions is evident in a multisite RCT conducted by Piera-Jiménez et al [47], who evaluated the economic impact of the same intervention implemented in 3 different countries: the Netherlands, Spain, and Taiwan. The study found that DTx led to QALY gains in the Netherlands but not in Spain or Taiwan, whereas cost savings were observed in Spain but not in the Netherlands or Taiwan.

### Methodological Characteristics of the Included Studies

With a mean CHEC score of 71% (SD 17.9%) across all 18 studies, the methodological rigor across the included studies was of moderate quality, ranging from an average of 90% (SD 8.2%) for RCT-based EE to 77% (SD 11.7%) for model-based EE to 53% (SD 6.7%) for nonrandomized study–based EE. In particular, the evaluations based on nonrandomized studies, all of which were funded by industry, adopted a payer-only perspective, which may be too narrow to broadly inform implementation decisions because it excludes direct patient out-of-pocket costs, indirect costs such as productivity loss, and other factors that can impact the long-term utility of an intervention. Furthermore, none of the EE based on nonrandomized studies performed an incremental analysis of costs and outcomes of the alternatives to DTx (eg, standard therapy). Finally, only 29% (2/7) of nonrandomized study–based EE performed sensitivity analysis, which is the best practice for quantifying uncertainty and testing the robustness of a study’s conclusions [50].

Another methodological deficit stems from the fact that although the majority of DTx interventions were reported to have significant impacts on costs and outcomes over a patient’s lifetime, most studies used a short time horizon to capture all or most clinical and economic impacts of the respective intervention. Specifically, the average time horizon of the RCT-based EE was 10.8 months, whereas that of the nonrandomized study–based EE was 17.8 months. Only 2 studies [37,45] adopted a lifetime horizon, and only 6 studies [37-39,44,45,47] included modeling decisions to extrapolate the outcome measures over time. This incongruity between the claimed lasting impacts of DTx and the limited time horizons over which they were evaluated implies that DTx should be assessed over longer periods [41,44,46,47]. In turn, determining the long-term economic effects of DTx and advancing understanding as to where their adoption may add value requires more comprehensive modeling [51].

Modeling can also ensure that trial populations reflect patient groups treated in real-world clinical practice, which is an important consideration because this review identified various biases in participant recruitment. Such biases might result in imbalances in the relevant baseline characteristics between patient groups, which can also hinder health equity considerations. As a case in point, in 11 studies [30-33,35,36,41-43,46,47], participants were required to have internet access, a smartphone or a tablet, the skills necessary to use a PC, medical insurance, or employment to participate—requirements that may limit the participation of members of marginalized groups or groups considered socioeconomically disadvantaged. In contrast, only 3 studies [32,43,46] addressed the ethical and distributional issues inherent in the implementation of digital technologies.

Across studies using the same perspective, disparities in the costs taken into account were also noted (Table 2). The importance of the time and expertise required for patient education on using and managing DTx technology [52] was only considered in 2 studies [46,47]. Similarly, only 1 study [40] factored in the ongoing maintenance costs of the DTx. Highlighting the criticality of taking stock of all costs, Lewkowicz et al [44] applied a societal perspective and reported that their model accounted for 61% of costs related to conventional treatment for low back pain when only direct costs were considered and for 81% when indirect costs were included, using a publicly available cost-of-illness study as a benchmark. Future DTx EE will therefore benefit from a more transparent, systematic, and exhaustive consideration of all the costs that implementing DTx interventions entails, including long-term health care costs that may not be directly disease- or intervention-related as per the Professional Society for Health Economics and Outcomes Research recommendations [50].

Altogether, the studied DTx interventions were found to be cost-effective in 9 (90%) of the 10 studies that performed a full EE [37,38,40-46] and cost saving in the remaining 8 studies that performed a partial EE [30-36,39]. In 5 (28%) of the 18 studies [42-45,47], the DTx interventions presented a trade-off between costs and effects (ie, intervention being more effective and more costly than the comparators). However, in 3 (60%) of these 5 studies [42,43,45], the highest ICERs obtained through sensitivity analysis fell below the willingness-to-pay threshold established in the countries in which they were performed, providing reassurance about their potential economic benefits.

The findings from this review indicate that DTx, at least in some use cases and local contexts, can be cost-effective and offer economic value to payers while simultaneously improving care for patients. However, consistent with the existing literature [49,51], qualitative deficits in methodology and significant potential biases in EE should be addressed going forward.

This review emphasizes the importance of adhering to established best practices and developing a robust, consistent methodological framework that incorporates the unique features that distinguish DTx interventions from conventional therapies or the current standard of care [49]. In the future, DTx EE analysis will need to adhere to local and international guidelines, use generalizable tools and metrics for enhanced comparability of the findings, and be both long-term focused and all-inclusive when factoring in value and cost. Such efforts are crucial for minimizing providers’, payers’, and patients’ uncertainties surrounding the adoption of DTx interventions.

### Limitations

Although we aimed to provide a comprehensive and systematic review of the economic value of clinical-grade mobile app–based DTx, there are several limitations to be acknowledged. First, only studies written in English were included. These studies were identified using a finite list of specific search terms; however, widely varying terminologies exist in the literature with reference to DTx, such as medical apps, digital therapies, or simply digital health technologies. As a result, it is possible that not all relevant studies assessing the economic impact of DTx may have been identified. Second, DTx interventions can be delivered through different modalities, including, but not limited to, virtual reality devices, mobile apps, web-based platforms, or a combination of these. To draw robust conclusions about mobile DTx as an emerging category of technologies in the clinical arena, this review focused exclusively on mobile app–based DTx and excluded multimodal DTx or those not primarily using a mobile app as the core delivery mechanism.

### Conclusions

This systematic review synthesizes the available evidence on the potential economic benefits of clinical-grade mobile app–based DTx as well as some of the qualitative deficits in DTx EE methodology, which can be used to guide future research on the subject. Specific areas that can benefit from more research and would further support market access decision-making and the adoption of DTx include evaluating DTx interventions in more diverse populations, across a greater variety of local contexts, and over longer time horizons.

## Appendix

Multimedia Appendix 1 Consensus Health Economic Criteria quality assessment.

Multimedia Appendix 2 Risk of bias for randomized clinical trial–based economic evaluations.

Multimedia Appendix 3 Risk of bias for nonrandomized studies–based economic evaluations.

Multimedia Appendix 4 Risk of bias for model-based economic evaluations.

## Abbreviations

CEA: cost-effectiveness analysis
CHEC: Consensus Health Economic Criteria
CUA: cost-utility analysis
DTx: digital therapeutics
EE: economic evaluation
ICER: incremental cost-effectiveness ratio
PRISMA: Preferred Reporting Items for Systematic Reviews and Meta-Analyses
QALY: quality-adjusted life year
RCT: randomized controlled trial
RoB: risk of bias
