# Supplementary figures and images for: Economic Evaluation Associated With Clinical-Grade Mobile App–Based Digital Therapeutic Interventions: Systematic Review
Source: J Med Internet Res. 2023 Aug 1;25:e47094. doi: 10.2196/47094 (PMC10427932; doi:10.2196/47094)

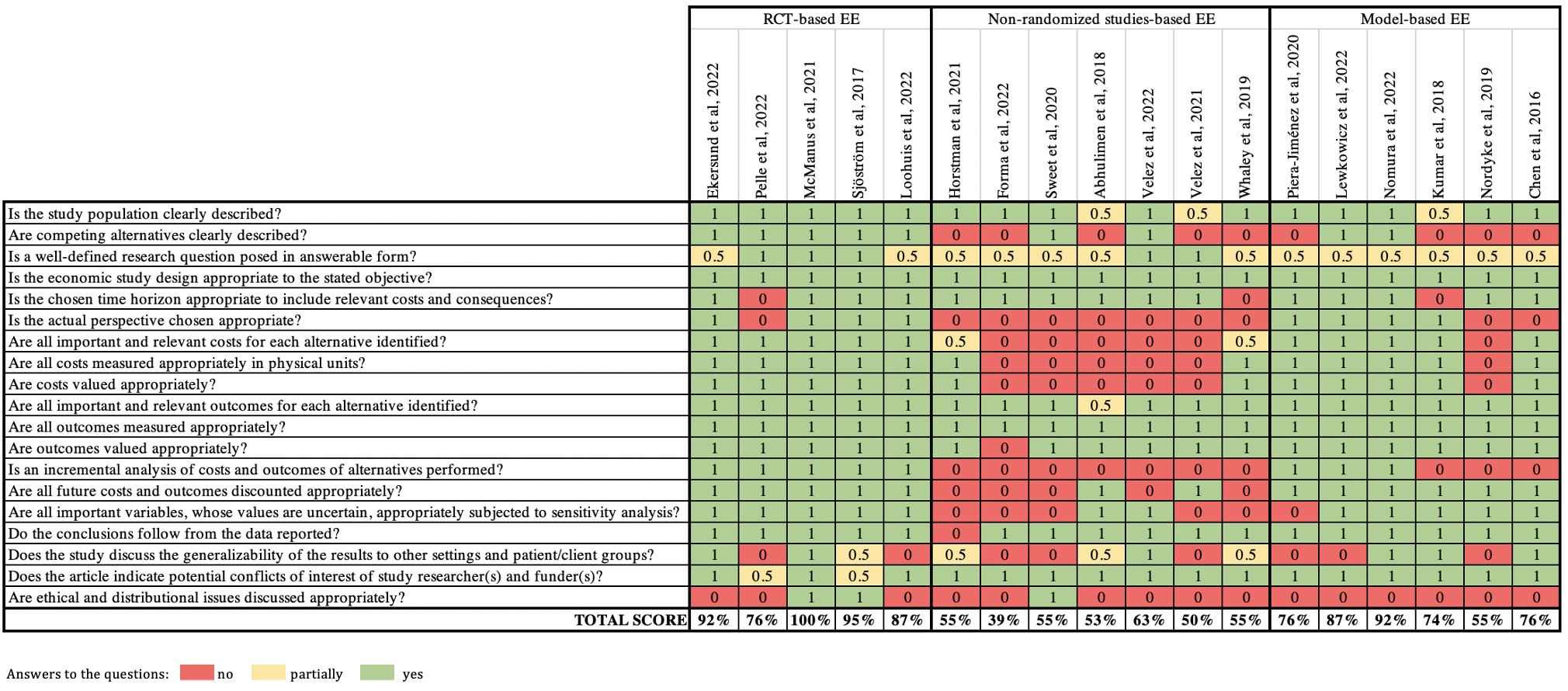

Supplement: Multimedia Appendix 1 [file jmir_v25i1e47094_app1.png]

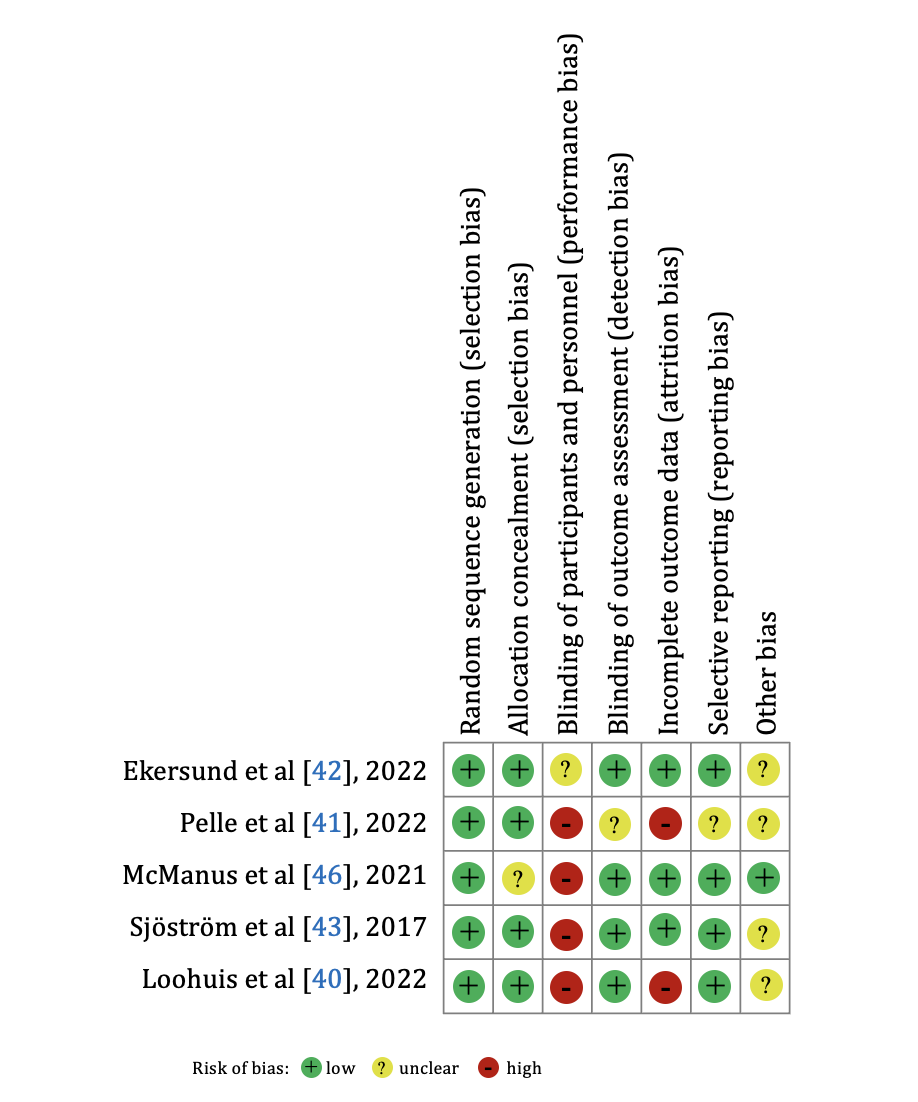

Supplement: Multimedia Appendix 2 [file jmir_v25i1e47094_app2.png]

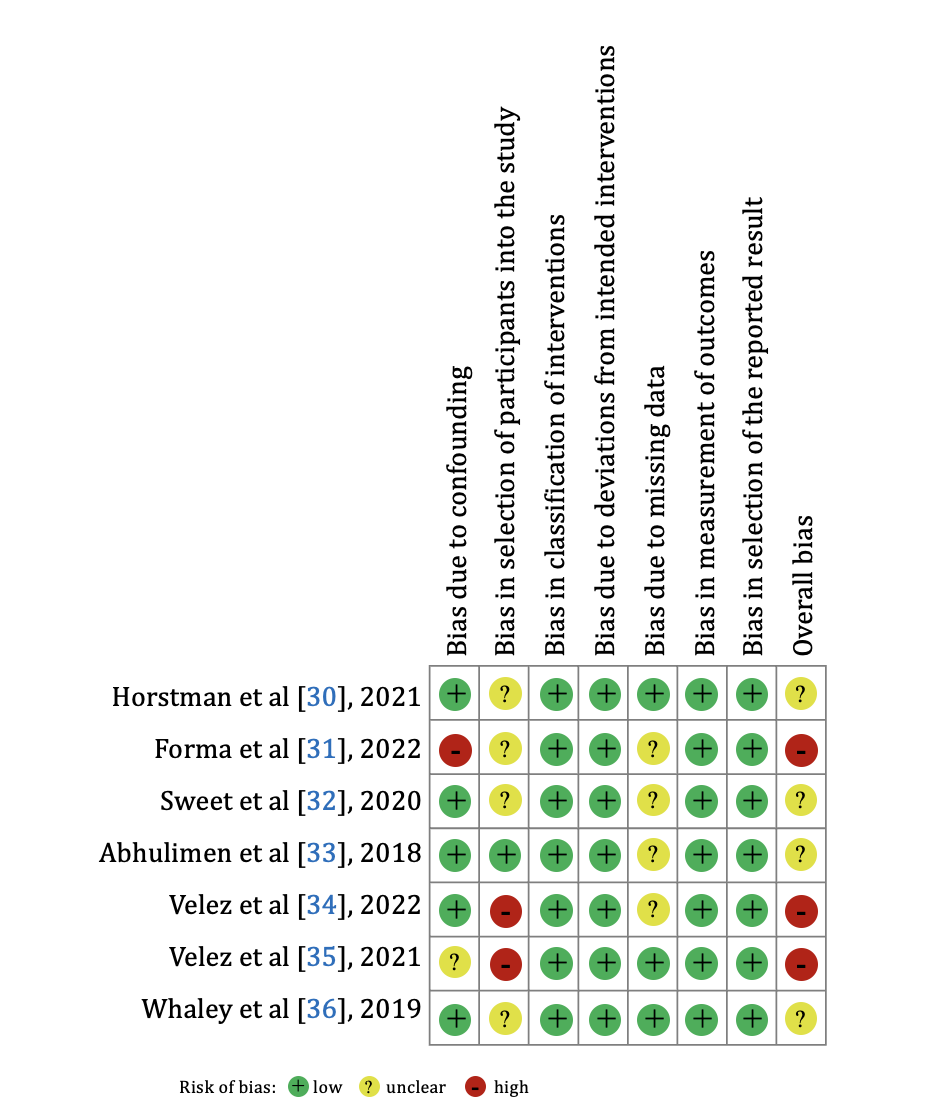

Supplement: Multimedia Appendix 3 [file jmir_v25i1e47094_app3.png]

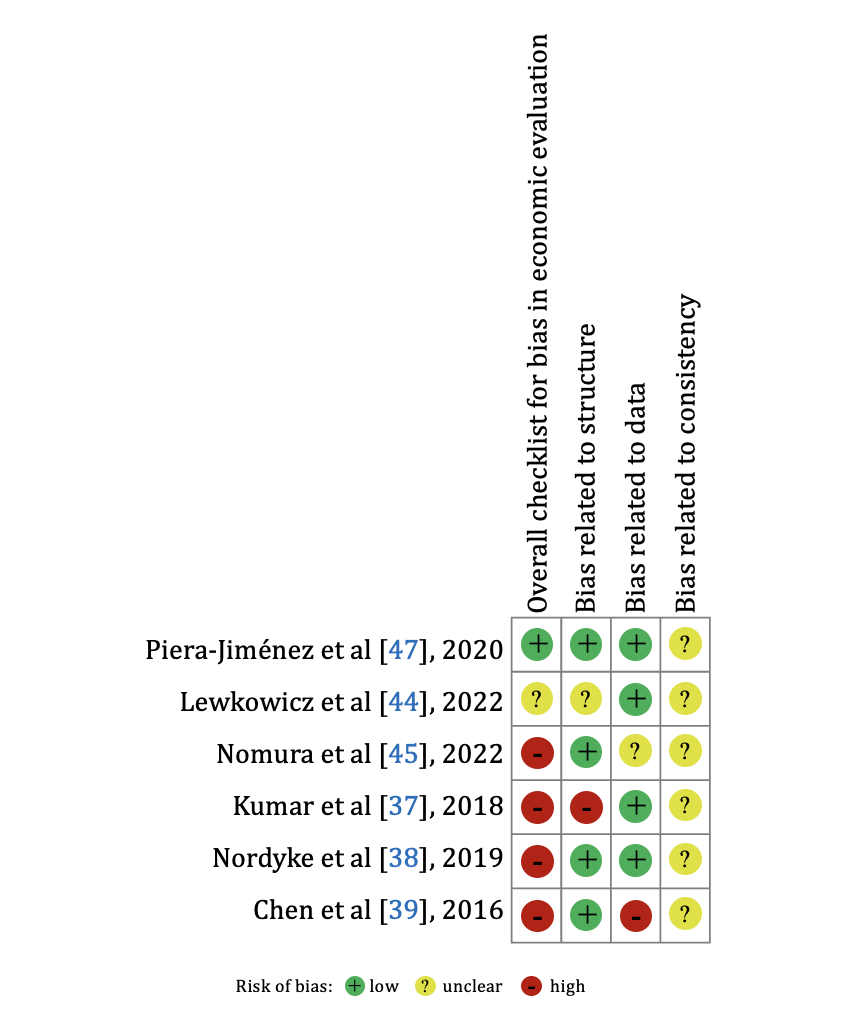

Supplement: Multimedia Appendix 4 [file jmir_v25i1e47094_app4.png]
